# Age-Related Alterations in Macrophage Distribution and Function Are Associated With Delayed Cutaneous Wound Healing

**DOI:** 10.3389/fimmu.2022.943159

**Published:** 2022-07-08

**Authors:** Christabel Thembela Dube, Yasmin Hui Binn Ong, Kelly Wemyss, Siddharth Krishnan, Tiak Ju Tan, Baptiste Janela, John R. Grainger, Matthew Ronshaugen, Kimberly A. Mace, Chin Yan Lim

**Affiliations:** ^1^ School of Biological Sciences, Faculty of Biology, Medicine and Health, University of Manchester, Manchester, United Kingdom; ^2^ Epithelial Epigenetics and Development Laboratory, ASTAR Skin Research Labs, Singapore, Singapore; ^3^ Lydia Becker Institute of Immunology and Inflammation, Faculty of Biology, Medicine and Health, Manchester Academic Health Science Centre, University of Manchester, Manchester, United Kingdom; ^4^ Skin Immunology Laboratory, ASTAR Skin Research Labs, Singapore, Singapore; ^5^ Skin Immuno-Monitoring Platform , Skin Research Institute of Singapore, Singapore, Singapore; ^6^ School of Medical Sciences, Faculty of Biology, Medicine and Health, University of Manchester, Manchester, United Kingdom; ^7^ Department of Biochemistry, Yong Loo Lin School of Medicine, National University of Singapore, Singapore, Singapore

**Keywords:** macrophages, inflammation, wound healing, myeloid cells, proliferation, ageing skin

## Abstract

Ageing-related delays and dysregulated inflammation in wound healing are well-documented in both human and animal models. However, cellular and molecular changes underlying this impairment in healing progression are not fully understood. In this study, we characterised ageing-associated changes to macrophages in wounds of young and aged mice and investigated transcriptomic differences that may impact the progression of wound healing. Full-thickness wounds created on the dorsum of C57BL/6J young and aged mice were excised on Days 3 and 7 post-wounding for analysis by immunohistochemistry, flow cytometry, and RNA sequencing. Our data revealed that macrophages were significantly reduced in aged wounds in comparison to young. Functional transcriptomic analyses showed that macrophages from aged wounds exhibited significantly reduced expression of cell cycle, DNA replication, and repair pathway genes. Furthermore, we uncovered an elevated pro-inflammatory gene expression program in the aged macrophages correlated with poor inflammation resolution and excessive tissue damage observed in aged wounds. Altogether, our work provides insights into how poorly healing aged wounds are phenotypically defined by the presence of macrophages with reduced proliferative capacity and an exacerbated inflammatory response, both of which are pathways that can be targeted to improve healing in the elderly.

## Introduction

The skin is an immunoprotective organ that functions to protect our internal environment from external insults. It is organised into the epidermal, dermal, and subcutaneous layers comprised of populations of specialised non-immune cells such as keratinocytes, fibroblasts, and adipocytes, respectively. Each of the skin’s main layers also consists of an interactive network of tissue-resident immune cells, such as macrophages, essential for the maintenance of homeostasis and skin integrity (1). Under steady-state, macrophages are reported as the most abundant immune cell type in the skin (2, 3). Here they perform immunosurveillance tasks and phagocytose invading pathogens upon injury or infection. Tissue-resident macrophages are distinguished into two main types, Langerhans cells, which reside within the epidermal layer, and dermal macrophages. Both Langerhans cells and dermal macrophages are deposited in the skin embryonically from fetal liver and yolk sac-derived precursors (4, 5). It has been proposed that in adult skin, Langerhans cells maintain their pools through self-renewal mechanisms whereas dermal macrophages are renewed and replaced by the recruitment of bone marrow-derived monocyte precursors (6–8).

Ageing is associated with a decline in the structural, and physiological function of the skin such as flattening of the dermal-epidermal junction, reduced elasticity, and low collagen density (9, 10). These ageing-related changes in the structure of the skin are linked to decreased cell replacement, impaired thermoregulation, increased susceptibility to infection, persistent low-grade inflammation as well as the reduced function of the resident cells such as macrophages (10–13). Altered function in the aged skin results in impaired barrier immunity and delayed cutaneous wound healing responses following injuries in the elderly (14, 15).

Cutaneous wound healing is the process of restoring the skin’s integrity following injury and occurs in four successive and overlapping phases: haemostasis, inflammation, proliferation and remodelling (16). Over the wound healing time course, distinct macrophage phenotypes with differential roles are found in each phase. Macrophages, both resident and recruited, have been shown to play important roles in the inflammatory and proliferative phase of wound healing (17, 18). Lucas et al. demonstrated that depletion of macrophages during the early inflammatory phase of healing in mice severely impaired wound closure, while depletion at the proliferative stage of healing resulted in excessive bleeding (18). These findings provide evidence that macrophages are a critical cell type in skin repair and highlight their phenotypic plasticity in response to the local wound healing microenvironment.

The progression of wound closure is delayed in aged skin, and poorly healing acute wounds in the elderly often develop into chronic wounds due to existing comorbidities such as diabetes and obesity (19). Chronic wounds are a major burden to the health care system due to the high costs of continuous treatment regimes in elderly patients, highlighting the need to improve wound healing progression in the elderly (20). Although poor healing in ageing skin is well-documented (21, 22), immune cell contributions underlying the delayed progression of healing have not been fully defined. Several reports have investigated the accumulation and distribution of macrophages, which play key roles in the initiation and resolution of inflammatory responses, in young and aged wounds (8, 14, 15, 21, 23). These studies yielded inconsistent, conflicting results due to the use of different models and quantification methods in the analyses. This has made interpretation and translation of findings to clinical applications more challenging. Further research is thus needed to establish the impact of ageing on macrophage behaviour and function in the wound healing process.

In this study, we characterised the effect of ageing on inflammatory cell distribution and function during full-thickness wound healing in young and aged C57BL6/J wild-type mice using more definitive approaches than previously utilised, such as high-parameter flow cytometry and RNA-sequencing of defined populations at critical time points in healing. Our data demonstrate extensive ageing-related impact on the number, distribution, and functional states of macrophages in the wounds. Aged wounds were found to contain reduced numbers of macrophages with diminished proliferative capacity and highly exacerbated inflammatory responses that likely contribute to impaired healing in aged mice.

## Materials and Methods

### Mice

Young (8-12 weeks) and aged (22-24 months) C57BL/6J wild-type female mice were housed with free access to food and water at the A*STAR Biological Resource Centre (*Singapore*) or Biological Services Facility at the University of Manchester (*Manchester, UK*). All procedures involving animals were approved by the A*STAR IACUC and the University of Manchester Ethical Review Committee under guidelines set by the Home Office and the Animals Scientific Procedures Act (1986). Mice at 8-12 weeks correspond to the equivalence of 15-30 years for humans, and at 22-24 months, the equivalence of humans above 60 years of age (24).

### Wounding and Tissue Harvesting

Mice were anaesthetised using 2% Isoflurane and two 10mm full-thickness excisional wounds were created on the dorsum using 100mm iris curved scissors (*World Precision Instruments*). 0.05mg/kg Buprenorphine was administered for pain relief. Mice were monitored daily to measure the wound diameter. The progression of healing was measured by calculating the percentage decrease in the original wounded area on Days 0, 3, and 7 post-wounding. Wounds were assessed for healing progression using the experimental wound assessment tool (EWAT) developed by Lima et al. (**Supplementary Table 1**) (25). The EWAT was adapted in this study for visual evaluation of unexcised wounds. Parameters, (**Supplementary Table 1**) including size of the wound area, changes in the surrounding skin due to inflammation, exudate amount, necrotic tissue amount and granulation tissue amount were assessed to evaluate the progression of healing in young and aged wounds. The total score was tabulated for all parameters assessed and high total EWAT scores were associated with poorer healing outcomes. Scoring was performed in a blinded manner where tissue samples were coded and scored without reference to any background information such as age group or day post-wounding (26). Tissues were harvested for further analyses from unwounded mice and on 3 or 7 days post-wounding by excising the wounded area and a 2mm perimeter of surrounding skin.

### Immunohistochemistry

5µm FFPE sections were deparaffinised in xylene and rehydrated in ethanol. Endogenous peroxidase activity was blocked using 2% hydrogen peroxide for 30 minutes (min). Enzymatic antigen retrieval was performed by incubating slides in 1% Trypsin-EDTA (*Sigma Aldrich*) for 30 min at 37°C. 16% normal goat serum (*Vector Laboratories*) was added to each section for 60 min at room temperature (RT) to block non-specific binding. Each section was then incubated at 4°C overnight in 100µl rat anti-mouse F4/80 primary antibody (*1:100, ab6640, Abcam*). Sections were washed in PBS for 10 min and incubated in 100µl of biotinylated anti-rat secondary antibody (*1:2000, BA-9400, Vector Laboratories*) for 60 min at RT. After washing with PBS, sections were incubated in 100µl of Vectastain ABC HRP stain (*Vector Laboratories*) for 30 min and then stained with 100ul of 3,3’-diaminobenzidine (*DAB Peroxidase (HRP) Substrate Kit, Vector Laboratories*). Sections were counterstained with Harris Haematoxylin (*Sigma Aldrich*), dehydrated in increasing concentrations of ethanol and 2 washes in xylene and mounted. Slides were imaged on a Pannoramic 250 Flash Slide scanner (*University of Manchester Bioimaging core facility*) and individual images were stitched together and viewed using CaseViewer (*3DHISTECH*). DAB-stained cells per mm^2^ were quantified using ImageJ software. For quantification of positive staining in wounds, scanned sections were divided 1mm^2^ fields of view and positive cells were counted using ImageJ software. The average number of positively stained cells per 1mm^2^ region was calculated for each mouse and 4 mice were assessed per group.

For haematoxylin and eosin staining (H&E), sections were deparaffinised as above and submerged in Harris haematoxylin for 2 min. Sections were rinsed with tap water and dipped into 0.25% acid alcohol. Each section was incubated in 95% ethanol for 30 seconds and then counterstained with Eosin Y (*Sigma Aldrich*) for 2 min. Slides were washed, dehydrated, and mounted as described above. To quantify reepithelialisation distance, the length of the epithelial tongue from the wound edge was measured from central wound sections and the average distance was calculated for each animal.

### Immunofluorescence

5µm FFPE sections were cut, deparaffinized, and rehydrated as described above. Heat-induced antigen retrieval was performed by incubating slides in 1X target antigen retrieval solution (DAKO, sodium citrate buffer, pH.6) for 20 min in a pressure cooker. Non-specific binding was blocked using 1% bovine serum albumin and 5% goat serum in PBS for 60 min, RT. Each section was incubated overnight at 4°C in 100ul of primary antibody cocktail containing rat anti-mouse F4/80 (1:100, ab6640, *Abcam*) and each of the following antibodies at a time: rabbit anti-IL1β (1:100; H-153, #*SC-7884*, *Santa Cruz*); rabbit anti- NFKB (p65, 1:100, D14E12, #*8242*, *Cell Signalling Technologies*); rabbit anti-Ki-67 (1:100, SP6, #*MA5-14520, Invitrogen*); rabbit anti-53BP1 (1:300, #*4937*, *Cell Signalling Technologies*). Secondary antibody incubation was carried out for 60 min, RT, using goat anti-rabbit Alexa Fluor 488 (1:400) and donkey anti-rat Alexa fluor 594 (1:400). Slides were washed in PBS, stained with DAPI solution for 15 min, and mounted using Prolong Gold antifade mountant (*Life Technologies*).

Immunofluorescence stained slides were imaged using the Olympus FLUOVIEW FV3000RS confocal microscope and associated software. Two fields of view (FOVs) per tissue section were captured from the wound centre at a resolution of, 1024 by, 1024 pixels using a 40X/1.30 Uplan FL N oil lens. All fields of view were imaged as z-stacks using the same laser and imaging settings for all three biological replicates in each group. Images were analysed using the ImageJ software. To measure the mean fluorescence intensity of IL1β or NFκB in F4/80^+^ cells, z-stack optical slices for each FOV were merged to obtain the average intensity projections. The F4/80 channel was then used to highlight each cell as a region of interest. The mean fluorescence intensity of the IL1β or NFκB channel was quantified in each F4/80^+^ cell. For F4/80 and Ki-67 or 53bp1 staining, the total number of double positive cells were manually counted using ImageJ.

### Isolation of Single Cells From the Skin and Wounded Tissues

Single cells were isolated from skin and whole wounds excised 3 and 7 days post-wounding. Prior to digestion, the adipose tissue was scraped and tissues were minced into small pieces. Tissues were agitated at 37° for 90 min in complete media containing 0.25mg/ml liberase (*Roche*) and 75 units/ml DNase 1 (*New England Biolabs*). Tissues were further dissociated twice using the m_muscle_01 protocol on a gentleMACS dissociator (*Miltenyi Biotec).* 0.5M EDTA was added to the suspension to stop the liberase reaction. Cells were passed through a 100µm cell strainer and washed twice with PBS supplemented with 3% foetal bovine serum.

### Flow Cytometry

For low-parameter flow cytometry, single cells isolated from young and aged wounds were stained in 100µl of antibody cocktail (**Supplementary Table 2**). The unfixed cells were analysed on a BD FACSAria (*BD Biosciences*). For high parameter flow cytometry, isolated single cells were surface stained with a 50µl of antibody cocktail in PBS (**Supplementary Table 3**) for 30 min at 4°C. Cells were washed twice in PBS and fixed in 2% paraformaldehyde (*Sigma Aldrich*) for 20 min at RT. Fixed cells were permeabilized with 0.5% saponin in PBS (*Sigma Aldrich*) for 10 min at 4°C. Cells were then intracellularly stained with 50µl of antibody cocktail (**Supplementary Table 3**) in 0.5% saponin for 60 min at RT. Cells were washed twice in 0.5% saponin and resuspended in PBS. OneComp eBeads (*Invitrogen*) were used for single stain compensation controls. Flow cytometry analyses were carried out on a BD LSRFortessa cell analyser using BD FACSDIVA software (*BD Biosciences*). Flow cytometry data were analysed on Cytobank (*Cytobank, California*). To minimise bias introduced by manually gated flow cytometry results, different populations were clustered using the viSNE algorithm, in Cytobank (27). Traditional gating strategies used to validate viSNE results are shown in **Supplementary Figure 1A**.

### Isolation and Culture of Bone Marrow-Derived Macrophages

To isolate mouse bone marrow cells, pelvic and femoral bones were dissected from young and aged wounded mice. Both ends of each bone were cut and bone marrow was flushed out using a 27G needle attached to a 10ml syringe filled with bone marrow growth medium (65% v/v High glucose Dulbecco’s Modified Eagle’s Medium – 4500mg/L glucose; 100units/ml-100µg/ml penicillin-streptomycin; 10% v/v foetal bovine serum (*Life Technologies*) and 20% v/v L-929 conditioned medium). Cells were then dissociated using a 19G needle and passed through a 70µm cell strainer.

Bone marrow cells were plated in 10cm non-coated dishes at a density of 1x10^6^ cells/ml and incubated at 37°C and 5% v/v carbon dioxide. Cells were fed on the third day by adding 7ml of growth medium. After growing for six days, cells were plated in 6 wells plates at a density of 0.5x10^6^ cells per well and grown for 24 hours. Cells were then classically activated for 24 hours by adding 1µg/ml lipopolysaccharides from *E.coli* and 100ng/ml Interferon-gamma (*Sigma Aldrich)*.

### Cell Cycle Analyses

Cell cycle analyses were carried out on LPS/IFNγ activated and non-activated bone marrow-derived macrophages (BMDMs) cells using the Click-iT Plus EdU flow cytometry assay kit (*Invitrogen*). 10µM EdU was added to young and aged BMDMs for 2hrs. Cells were harvested and washed with 1% BSA in PBS. Cells were then fixed using the Click-iT fixative and permeabilized with Click-iT permeabilisation and wash reagent. Click-iT EdU was detected according to the manufacturer’s instruction. FxCycle Violet dye (*Invitrogen*) was used to stain cells for DNA content. EdU labelled cells were then analysed by flow cytometry using a BD LSRII flow cytometer. Data analysis was carried out on Cytobank.

### RNA Extraction, cDNA Synthesis, and qRT-PCR From BMDMs

1ml of TRIzol reagent (*Invitrogen*) was directly added to plates containing young and aged activated and non-activated BMDMs. Total RNA extraction was carried out using the RNeasy mini kit according to the manufacturer’s instructions. RNA concentrations were assessed using the NanoDrop, 8000 (Thermo Fisher Scientific, Waltham*, MA*). 250ng of cDNA was synthesised using the High capacity cDNA Reverse Transcription kit (*Thermo Fisher Scientific*) according to the manufacturer’s instructions.

For quantitative real-time polymerase chain reaction (qRT-PCR), 2ul of cDNA was added to 5µl PowerUp SYBR Green Master Mix (*Applied Biosystems*), 2.8µl of water and 0.2µl of forward and reverse primer mix (**Supplementary Table 4** for primer sequences of *Cdk1* and *Ccnb1*). qRT-PCR was performed on a QuantStudio5 Real-Time PCR System (*Applied Biosystems*). Relative gene expression levels were normalised to the reference genes *Actb* and *Tbp.*

### RNA Extraction, cDNA Synthesis and qRT-PCR: FFPE Fixed Whole Wounds

To extract total RNA from FFPE whole wound tissue blocks, 6 20µm serial sections from young or aged Day 3 wound samples were placed in 1.5ml RNAse-free microcentrifuge tubes and deparaffinised in 1ml of xylene at 50°C for 3 minutes. Samples were washed twice in 100% ethanol and then treated in 300µl of 1X buffer (20mM Tris-HCl pH 8, 1mM calcium chloride, 0.5% SDS) containing 500µg/ml proteinase K (*Roche*) at 55°C for 16 hours. After proteinase K digestion, RNA was isolated by adding 1ml of TRIzol reagent (Invitrogen) to each sample for 5 min at RT. 0.2ml of chloroform was added for phase separation. Samples were centrifuged at, 12000xg for 15 min and the aqueous phase was carefully transferred to a new microcentrifuge tube containing 600µl of isopropanol and 10µg of Glycoblue coprecipitant (Invitrogen). Tubes were incubated at RT for 10 min and then centrifuged at, 12000xg for 20 min. Each pellet was washed in 1ml of ice-cold 70% ethanol three times and centrifuged at, 7500xg for 5 min. Sample pellets were allowed to air dry for 10 min and then dissolved in 30µl of nuclease-free water. RNA was quantified and assessed for purity using a Nanodrop ND-1000 spectrophotometer and A260/280 ratios were between 1.80-2.00.

The isolated RNA was treated with TURBO DNA-free kit (*Invitrogen*) and used for cDNA generation using a Tetro cDNA Synthesis Kit (*Bioline, Memphis, TN*) according to the manufacturer’s instructions. For qRT-PCR, 1µl of cDNA and its negative control (minus reverse transcriptase) was added to a mixture of 5µl 2X TaqMan fast universal PCR master mix, 3.5µl nuclease-free water and 0.5µl of 20X TaqMan gene expression assay (*Adgre1*, Mm00802529_m1 (amplicon=92bp); Hist2aa2 (amplicon=57bp), Mm00501974_s1; *Applied Biosystems*). Relative gene expression levels (relative to Histone 2A, reference gene) were compared between young and aged samples.

### RNA Extraction, cDNA Synthesis and qRT-PCR: Sorted Tissue Macrophages

RNA lysates were thawed on ice and total RNA was extracted using the RNeasy Plus Micro Kit (*Qiagen*) according to the manufacturer’s instructions. cDNA synthesis was carried out using the High capacity cDNA Reverse Transcription kit (*Thermo Fisher Scientific*) as previously described. All cDNA were preamplified using the Prelude PreAmp Master Mix (*TakaraBio)*. 1µl of cDNA was added to 25µl preamp master mix, 5µl of primer pool (500nM stock primer concentration, **Supplementary Table 5**) and 19µl of water. The reaction mix was cycled 14 times according to the manufacturer’s recommendations. qRT-PCR was carried out using PowerUp SYBR Green Master Mix (*Applied Biosystems*). List of primers used is shown in **Supplementary Table 4**. Relative gene expression levels were normalised to the reference gene *Actb.*

### Fluorescence-Activated Cell Sorting (FACS)

To sort out CD45^+^, CD11b^+^, CD64^+^, F480^+^ live macrophages (gating strategy and percentage live dead, **Supplementary Figures 1B–C**) for RNA sequencing, cells from Day 3 young and aged wounds were stained in 100µl of antibody cocktail (**Supplementary Table 2**). Unfixed cells were fluorescence-activated cell sorted on a BD FACSAria cell sorter (*BD Biosciences*). The panel used did not have an exclusion parameter for Ly6G^+^ cells, therefore, sorted cells may contain a minor spillover of neutrophils. Cells were directly sorted into RNeasy Buffer RLT lysis buffer (*Qiagen*) supplemented with 1% β-Mercaptoethanol. Lysates were homogenised using QIAshredders (*Qiagen*) according to the manufacturer’s manual and flash-frozen for later use.

### Bulk RNA Sequencing

Total RNA samples from young and aged Day 3 sorted wound macrophages were submitted to an NGS service provider for mRNA sequencing (Novogene, Singapore). Library construction and quality evaluation were performed by Novogene. Pooled libraries were sequenced on a Novaseq6000 platform with a 150bp paired-end read length at an average depth of 50 million reads per sample.

Sequenced data were processed using the Partek Flow genomics analysis software (*Partek*). Raw reads were aligned using Star 2.7.3a following pre-alignment quality check. Transcripts were quantified to mm10 mouse genome Ensemble Transcripts release 102. Raw gene counts were normalised and differential expression analysis was carried out using DESeq2 with the Benjamini Hochberg false discovery rate multiple testing. We applied a threshold cut-off of fold change > ± 2, *p*-value < 0.05 and a false discovery rate < 0.05. Principal component analyses, hierarchical clustering and KEGG pathway analysis were all performed in Partek Flow genomic suite. Further functional analyses were carried Gene Set Enrichment Analysis Software (GSEA, *Molecular Signatures Database*) and on the STRING database.

### Statistical Analyses

All statistical analyses were carried out on GraphPad Prism 9. Unpaired Student’s t-tests (two-tailed) were used to compare significant differences between two groups. Two-way ANOVA with a Bonferroni multiple comparisons test was used to compare three or more groups of data. Data are shown as mean ± SD and the level of significance was defined as *p* < 0.05 unless otherwise stated.

## Results

### Dermal Macrophages Are Reduced in Aged Mouse Skin

**Figure 1:**
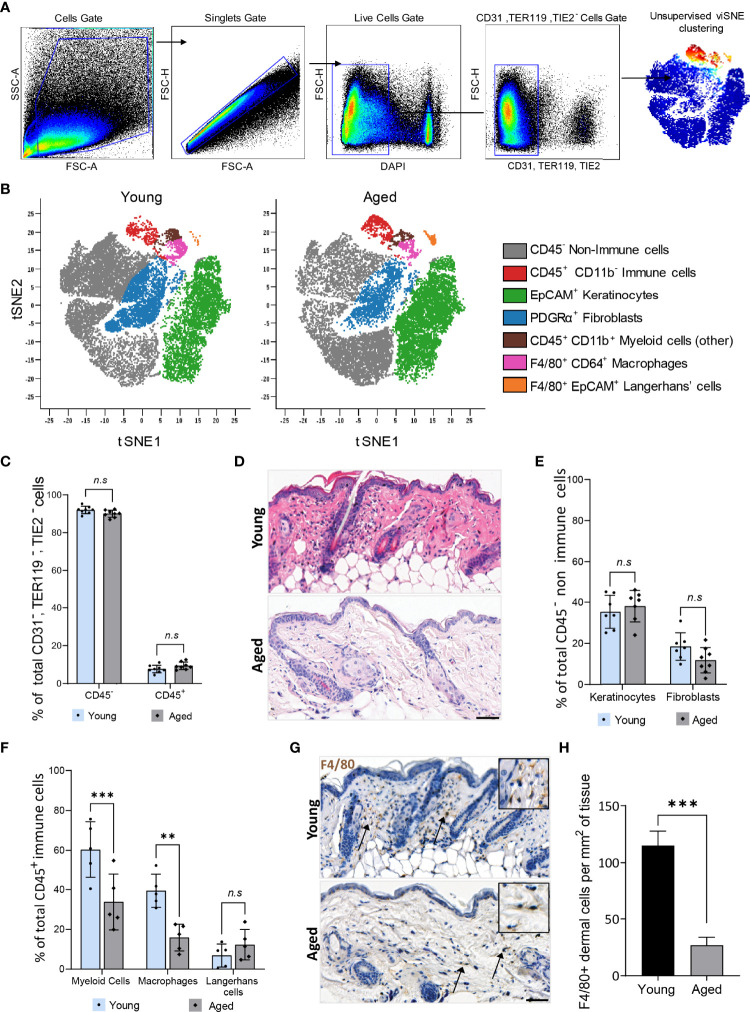
Dermal macrophages are significantly reduced in aged skin. **(A)** Flow cytometry gating strategy used to isolate live single CD31^-^, TER119^-^, TIE2^-^ cells from young and aged murine skin. **(B)** Overlaid viSNE map illustrating 7 distinct populations in young and aged skin. **(C)** Mean percentage population of total CD45^+^ immune and CD45^-^ non-immune cells in young and aged skin. n=8 mice per group. **(D)** Haematoxylin and Eosin (H&E) staining of young and aged murine skin. Scale bar= 50µm. **(E)** Percentage population of EpCAM^+^ keratinocytes and PDGFRα^+^ fibroblasts. n=8 mice per group. **(F)** Percentage of CD11b^+^ myeloid cells, F4/80^+^ CD64^+^ macrophages and F4/80^+^ EpCAM^+^ Langerhans cells. n=5 mice per group. Data in **(C, E, F)** were analysed by two-way ANOVA with a Bonferroni multiple comparisons test. **p=0.003, ***p<0.001, *n.s*, *not significant*. **(G)** Micrographs of the distribution of F4/80^+^ macrophages in dermal murine skin. Scale bar= 50µm. **(H)** Average number of F4/80^+^ dermal macrophages per mm^2^ of young and aged skin tissue. n=4 mice per group. ***p<0.001, unpaired t-test. Bars show mean ± SD.

Studies in humans revealed alterations in the function but not the global distribution of resident cell types such as fibroblasts, keratinocytes, and total immune cells in aged skin (28, 29). We corroborated these findings on cells isolated from skin tissues of young (12 weeks old) and aged (22-24 months old) C57BL/6 mice. Live, single CD31^-^TER119^-^TIE2^-^ cells were pre-gated and subjected to unsupervised Cytobank viSNE (**Figure 1A**). Seven clusters of resident skin cells were identified using six markers (**Figure 1B**, **Supplementary Table 2**, **Supplementary Figure 2**). No ageing-related differences in the proportion of total CD45^-^ non-immune cells (90% ± 1.9%) and CD45^+^ immune cells (10% ± 1.9%) were observed (**Figure 1C**). Although aged mouse skin histologically exhibited a less cellular dermis characterised by disorganised connective tissue (**Figure 1D**), the distribution of CD45^-^PDGFRα^+^ fibroblasts and EpCAM^+^ keratinocytes was similar between aged and young tissues (**Figure 1E**). Hence, consistent with findings in humans, ageing does not alter the total proportion of the major non-immune cell types in mouse skin. CD45^+^ immune cell subtypes were, however, differentially distributed in an age-dependent manner. CD11b^+^ myeloid cells constituted 60.3% ± 14.1% of total CD45^+^ cells in young tissues which decreased to 33.8% ± 14.0% in aged skin (**Figure 1F**). Of the CD11b^+^ myeloid cells, a prominent 60% decrease (young=39.4 ± 8.3%, aged=15.90 ± 6.7%) in F4/80^+^CD64^+^ macrophages was observed in aged skin while F4/80^+^ EpCAM^+^ Langerhans cells remained unchanged (**Figure 1F**). Immunohistochemical analyses of young and aged tissues showed similarly that aged tissues had 80% fewer macrophages compared to young skin (**Figures 1G–H**). These results indicate that a reduced number of dermal macrophages is a characteristic feature of aged skin.

### Slow-Healing Wounds in Aged Mice Have Reduced Numbers of Macrophages

**Figure 2:**
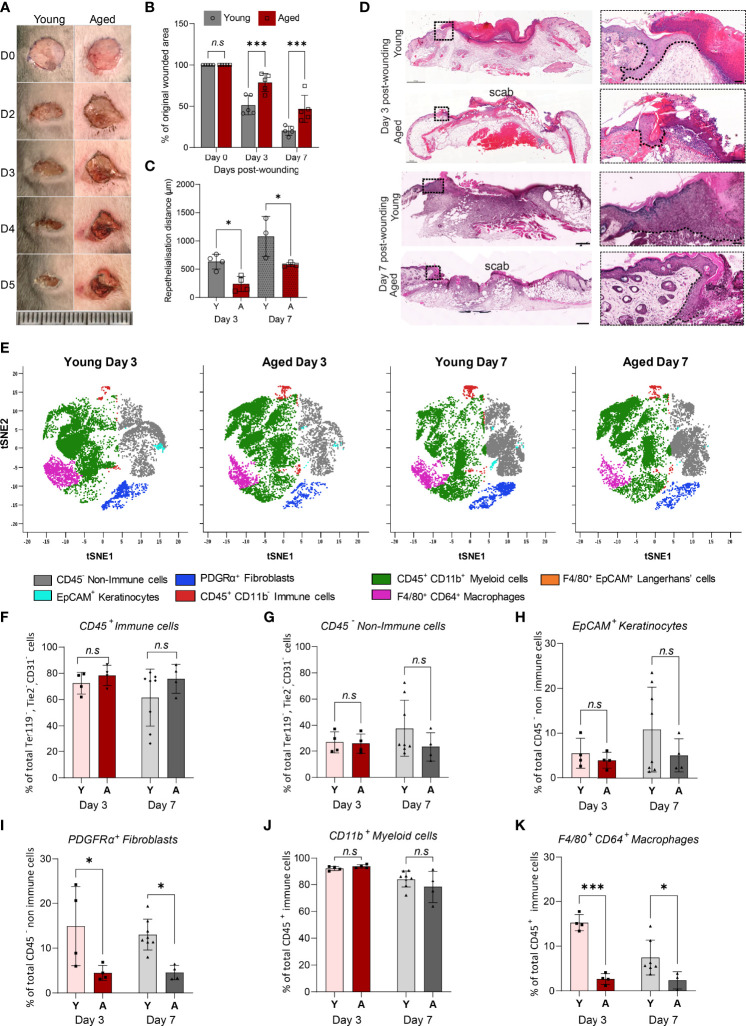
Delayed healing wounds in aged mice exhibit a reduced proportion of macrophages compared to young wounds. **(A)** Photographic images of full-thickness wounds in young and aged mice over a 5-day healing period (Scale bar=25mm). **(B)** Percentage change in wounded area proportional to original wound size in Day 0, 3 and 7 wounds. Data are plotted as mean ± SD. n=5 mice per group ***p<0.001, two-way ANOVA with a Bonferroni multiple comparisons test. **(C)** Reepithelialisation distance calculated by measuring the length of the epithelial tongue from the wound edge in young and aged Day 3 and 7 central cross-sections. *p<0.05, one-way ANOVA with Bonferroni multiple comparisons. Dashed lines trace the base of the migrating epithelial tongue. **(D)** H&E staining of young and aged wounds on Days 3 and 7 post wounding. Scale bar= 500µm. **(E)** viSNE analysis of, 10000 live single CD31^-^, TER119^-^, TIE2^-^ cells per group using 6 markers. Overlaid viSNE plots map 7 distinct populations identified in young and aged wounds 3 and 7 days post-wounding. **(F–K)** Percentage population of CD45^+^ immune cells, CD45^-^ non-immune cells, EpCAM^+^ keratinocytes, PDGFRα^+^ fibroblast, CD11b^+^ myeloid cells, and F4/80^+^ CD64^+^ macrophages in young and aged mice 3 and 7 days post-wounding. Bars show mean ± SD. n is a minimum of 4 mice per group. *p<0.05, ***p<0.001, *n.s, not significant*, two-way ANOVA with a Bonferroni multiple comparison test. Y=young, A=aged.

To characterise cellular composition changes that underlie ageing-associated alterations to injury response and healing, full-thickness wounds were created on the dorsum of young and aged WT mice. The rate of wound closure over a 7-day healing time-course was significantly slower in aged mice (**Figures 2A, 2B**). Day 3 and Day 7 wounds in the aged mice had reduced levels of re-epithelialization (**Figures 2C, 2D**). Unsupervised viSNE clustering of cells isolated from Day 3 and Day 7 wounds on young and aged mice revealed key differences in the cellular composition of non-wounded and wounded skin, as well as ageing-related changes in wounds (**Figure 2E**, **Supplementary Figure 3**). In both Day 3 and Day 7 wounds, CD45^+^ immune cells constituted the majority of total cells and the ratios of immune and non-immune cells in the wounds were not significantly different in young and old mice (**Figures 2F, G**). While the proportions of EpCAM^+^ keratinocytes were not significantly changed, PDGFRα^+^ fibroblasts were reduced by 70% and 65% in Day 3 and Day 7 wounds, respectively, in aged mice compared to the corresponding wounds in young animals (**Figures 2H, 2I** *)*.

Unlike in non-wounded skin, age-related differences were not observed in the proportions of myeloid cells, which made up 80-90% of total CD45^+^ immune cells, in the wounds (**Figure 2J**). However, distinct ageing effects in the relative abundance of F4/80^+^CD64^+^ macrophages were observed. In the young Day 3 wounds, F4/80^+^CD64^+^ macrophages constituted more than 15% ± 1.8% of total CD45^+^ cells but the proportion of macrophages was significantly lower (2.7% ± 1.2%) in the aged wounds (**Figure 2K**). As healing progressed, the proportion of F4/80^+^CD64^+^ macrophages decreased to 7.5% ± 3.9% of total CD45^+^ cells in the young Day 7 wounds but was not altered in the corresponding aged wounds. These results suggest that while immune cell responses to wounding were initiated in both young and aged mice, macrophage accumulation in response to injury is significantly altered in the aged mice in the early stages and persists through the healing process.

### High Parameter Mapping of Myeloid Cell Subtypes in Young and Aged Day 3 Wounds

We further analysed the composition of myeloid populations to assess if ageing affected the distribution of cells other than macrophages in Day 3 wounds. We focused on Day 3 wounds as this time-point exhibited the largest age-related differences in cellular distribution and represents the peak of inflammation following injury. Day 3 is thus a critical time-point in healing as appropriate regulation of inflammation at this stage is essential for the formation of a microenvironment favourable for optimal progression of wound closure (30).

**Figure 3:**
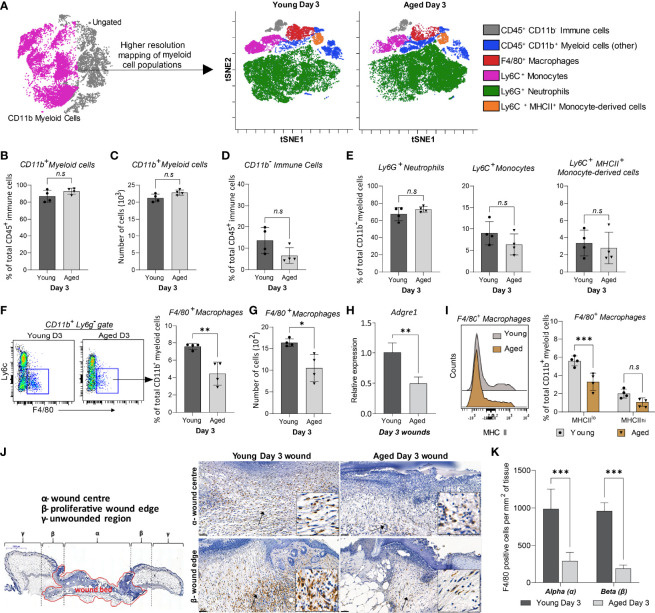
High-resolution mapping of myeloid cells shows an ageing-associated differential distribution of macrophages 3 days post-wounding. **(A)** Overlaid viSNE plots illustrate the distribution of 6 myeloid subtype populations., 25000 cells per group were analysed by viSNE. **(B-C)** Quantification of proportions and the total number of CD11b^+^ myeloid cells. **(D-E)** Proportion of CD11b^-^ immune cells, Ly6G^+^ neutrophils, Ly6C^+^ monocytes, and Ly6C^+^ MHCII^+^ monocyte-derived cells in young and aged days wounds. **(F–G)** Percentage and number of F4/80^+^ macrophages in young and aged wounds. Bars show mean ± SD, n=4 mice per group. **(H)** Relative mRNA expression of *Adgre1* (F4/80) in young and aged wounds. **(I)** Percentage of MHCII^lo^ and MHCII^hi^ macrophages subsets in young and aged wounds. **(J)** Representative micrographs showing the distribution of F4/80^+^ macrophages in young and aged tissues 3 days post-wounding per wound region (quantification method adapted from Borue et al. (31). **(I)** Number of F4/80^+^ macrophages in the α-wound centre and the β-wound edge per mm^2^ of tissue. n=4 mice per group. Data were analysed by unpaired t-test **(B-H)** and two-way ANOVA with Bonferroni multiple comparison test (I, K). Bars show mean ± SD *p<0.05, **p<0.01, ***p<0.001, *n.s*, *not significant*.

ViSNE analyses on gated live, single, CD3^-^NK-1.1^-^B220^-^CD19^-^CD45^+^ cells identified five distinct myeloid subpopulations (**Figure 3A**, **Supplementary Figure 4**). Total CD11b^+^ myeloid and CD11b^-^ cell populations were consistent in both young and aged Day 3 wounds, confirming our earlier findings (**Figures 3B-D**, **2J**). The distribution of Ly6G^+^ neutrophils, Ly6C^+^MHCII^-^ infiltrating monocytes, and Ly6C^+^MHCII^+^ monocyte-derived cells was unchanged between the young and aged mice (**Figure 3E**). F4/80^+^ macrophages were the only myeloid cells to exhibit an age-related alteration to their distribution. The total number and proportion of macrophages in the wounds were reduced by 40% in the aged compared to young Day 3 wounds, concomitant with decreased expression of *Adgre1* (encoding F4/80) in the aged Day 3 wounds (**Figures 3F–H**). Upon further analysis, we found that F4/80^+^ macrophages in wounds can be further distinguished into F4/80^+^MHCII^hi^ and F4/80^+^MHCII^lo^ subtypes. Notably, aged wounds had a significantly lower proportion of F4/80^+^MHCII^lo^ but not F4/80^+^MHCII^hi^ macrophages (**Figure 3I**). Immunohistochemical analysis of whole Day 3 wounds confirmed that F4/80^+^ macrophages were more than 50% lower in the centre and proliferative edges of the wounds in aged compared to young mice (**Figures 3J, K**). Altogether, our data demonstrate that macrophages are the only significantly reduced myeloid subtype in aged wounds, whereas their distribution, especially of the F4/80^+^ MHCII^lo^ macrophages, appears to be most impacted upon ageing.

### Transcriptomic Analyses Reveal Ageing-Related Impairment of Macrophage Function

**Figure 4:**
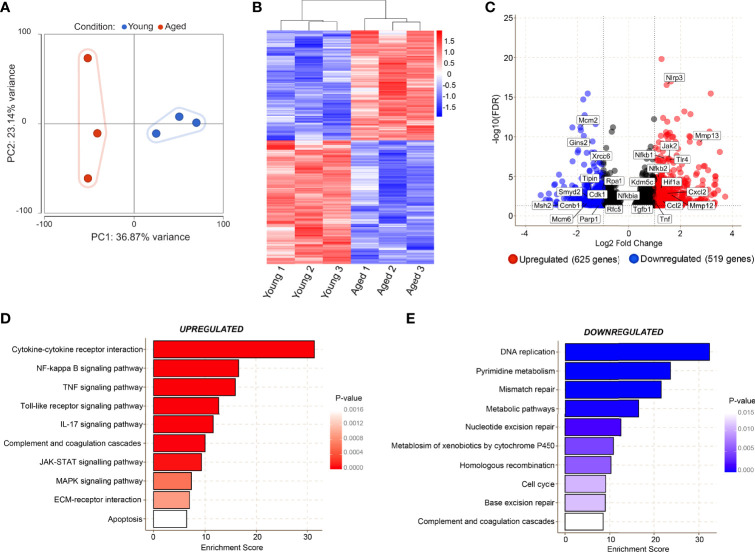
Transcriptomic analyses reveal age-related alterations in key molecular pathways in macrophages during the inflammatory phase of wound healing. **(A)** Bulk RNA sequencing was carried out on FACS sorted macrophages from young and aged wounds 3 days post-wounding. Principal component analysis (PCA) plot showing variation between 3 young (blue) and 3 aged (red) macrophage samples sorted from wounds. The plot models 60% of the total variance. **(B)** Hierarchical-clustering heatmap by samples of, 1144 differentially expressed genes in young and aged macrophages. **(C)** Volcano plot of differentially expressed genes between young and aged macrophages. A cutoff FDR >0.05; Log2 Fold Change > ± 1 was used. 625 genes were upregulated whereas 519 were downregulated. **(D, E)** Top enriched KEGG pathways from genes upregulated (*red*) and downregulated (*blue*) in aged wound macrophages (*p<0.05*).

RNA-sequencing on CD45^+^CD11b^+^F4/80^+^CD64^+^ macrophages (manual gating strategy and percentage live dead values are shown in **Supplementary Figure 1B, C**) from young and aged Day 3 wounds was performed to investigate the molecular alterations in these cells. Principal component analysis revealed high similarity in macrophages isolated from young wounds, while the aged macrophages exhibited high inter-sample variability along PC2 (**Figure 4A**). Genes associated with the inflammatory macrophage identity such as *Fth1*, *Lyz2*, *Cd14* and *Cd74*, were highly expressed in both young and aged populations, validating the purity of the sorted cells., 1144 genes were found to be differentially expressed by 2-fold or greater between young and aged Day 3 wound macrophages, of which 625 genes were upregulated and 519 downregulated in the aged samples (**Figures 4B, C**, **Supplementary Material 1**). KEGG pathway analysis of the differentially expressed genes revealed molecular alterations to major functional pathways in the aged wound macrophages. Pro-inflammation-related pathways such as cytokine-cytokine receptor interaction; NF-κB, TNF, and JAK-STAT signalling pathways; and apoptosis were up-regulated in the aged macrophages (**Figure 4D**). These cells exhibited a hyper-inflammatory transcriptome signature in which the expression of key inflammation-associated genes such as *Il1b*, *Il6*, *and Nfkb1* was highly elevated (**Figure 4C**). Compared to young macrophages, genes involved in DNA replication, repair, and cell cycle pathways, including subunits of the minichromosome maintenance complex (MCM), were significantly down-regulated in the aged macrophages (**Figures 4C, E** **)**. These results reflected a perturbed transcriptomic profile in aged macrophages that is indicative of reduced proliferative capacity alongside a triggered inflammatory cell state.

### Proliferation Related Pathways Are Intrinsically Altered in Aged Macrophages

**Figure 5:**
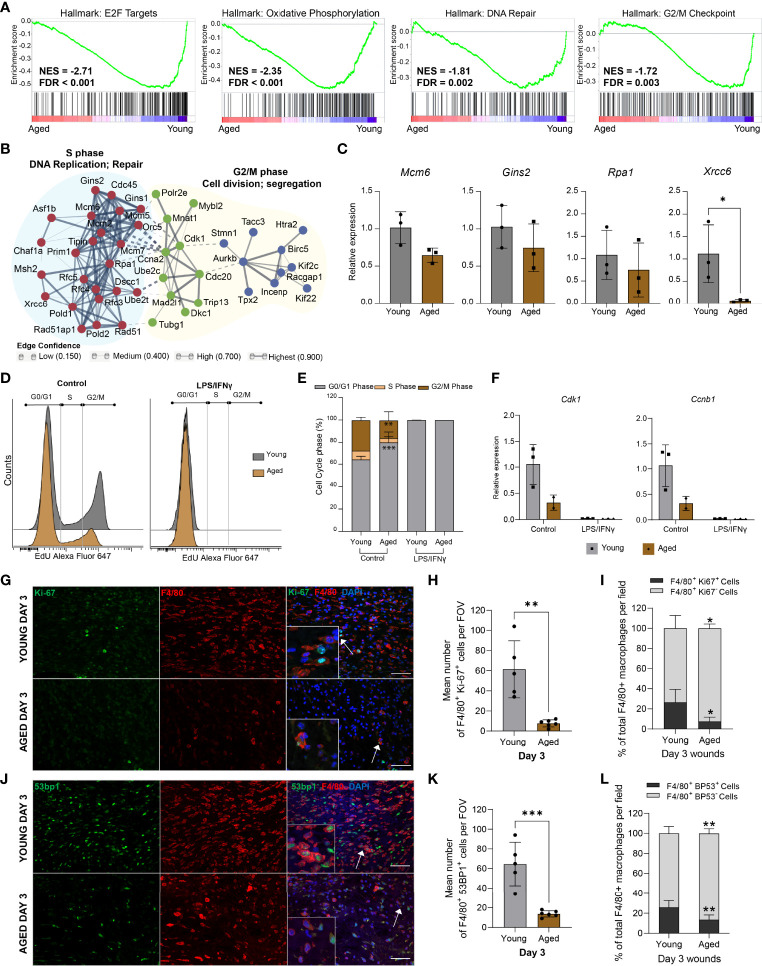
Aged macrophages exhibit altered cell cycle profiles **(A)** GSEA plots of top downregulated cell cycle-related pathways in aged wound macrophages. The normalised enrichment score (NES) and the false discovery rate q-value (FDR) are shown. **(B)** Protein-protein interaction networks of differentially expressed leading-edge genes between young and aged mice created in STRING. **(C)** Relative expression of DNA repair-related genes *Mcm6, Gins2, Rpa1*, and *Xrcc6* in macrophages sorted from young and aged Day 3 wounds. Expression levels were normalised to *Actb*. n=3 mice per group, unpaired t-test. ***(*D*)*** Flow cytometry histograms of cell cycle analysis in non-activated (control) and activated BMDMs (24hrs LPS+ IFNγ) from young and aged wounded mice. BMDMs were treated with EdU for 2hrs before analysis. **(E)** Quantification of cell cycle analyses by phase in young and aged BMDMs. n=3 mice per group, two-way ANOVA with a Bonferroni multiple comparison test. **(F)** Expression of *Cdk1* and *Ccnb1* in young and aged non-activated and activated BMDMs. Expression levels were normalised to the average of 2 housekeeping genes *Actb* and *Tbp*. All groups were normalised to young control. n=2-3 mice per group. **(G, J)** Representative micrographs of F4/80 + Ki-67 **(*G*)** and F4/80 + 53BP1 **(J)** staining distribution in young and aged Day 3 wounds. Scale bar= 50µm. **(H, K)** Mean number of cells that were double-positive for F4/80 and Ki67 **(*H*)** or 53BP1 **(*K*)** in young and aged Day 3 wounds per field of view. **(I, L)** Percentage of F4/80^+^ Ki67^+^ or F4/80^+^ 53BP1^+^ cells as a proportion of total F4/80^+^ cells present in young and aged Day 3 wounds. Bars show mean ± SD, n= a total of 5-6 FOVS taken from the wound centre from 3 mice per group (2 FOVs per mouse). *p<0.05, **p<0.01, ***p<0.001, unpaired t-test.

Unbiased gene set enrichment analysis (GSEA) revealed distinct associations of the young macrophage transcriptome with Hallmark gene sets (32), for E2F targets, G2/M checkpoint, DNA repair, and oxidative phosphorylation, indicating the down-regulation of proliferative processes in aged macrophages (**Figure 5A**). The leading edge genes of the “E2F targets”, “DNA repair”, and “G2M checkpoint” gene sets were used to construct a functional association network of the most down-regulated genes using the STRING database (33, 34). Markov clustering revealed the down-regulation of three linked gene networks correlated to DNA synthesis and repair, cell division, and chromosome segregation (**Figure 5B**). Differential expression of downregulated DNA repair and cell cycle genes was assessed by qPCR analysis on macrophages isolated from young and aged Day 3 wounds (**Figure 5C**).

To ascertain the effect of these ageing-associated transcriptomic changes on the proliferation potential of the macrophages, we derived macrophages from the bone marrow (BMDMs) of young and aged wounded mice and analysed the cell cycle profiles of non-activated and LPS/IFNγ-activated BMDMs. Consistent with previous studies (35), LPS treatment induced a block at the G1-S transition and arrested the proliferation of BMDMs derived from young mice (**Figures 5D, E** **)**. Although aged BMDMs were reported to be less responsive metabolically to LPS stimulation (36), these cells were also arrested in the G0/G1 phase by LPS treatment. More notably, we observed 15.8% more non-activated BMDMs derived from aged mice in the G0/G1 phase compared to young BMDMs (**Figures 5D, E**). Evaluation of key mitotic checkpoint genes such as *Cdk1* and *Ccnb1* indicated a reduction of these genes in the aged non-activated BMDMs (**Figure 5F**).

These results suggest that macrophages, both from wounds or derived from bone marrow, exhibit an ageing-related cell-intrinsic reduction in proliferative potential, accompanied by the down-regulation of genes involved in chromosomal DNA metabolism and repair. We sought to corroborate these findings by assessing the macrophages within their native environment in the Day 3 wounds of young and aged mice. Double immunostaining for the macrophage marker F4/80^+^ and the proliferation marker Ki67 was carried out to determine the proportion of proliferative macrophages in the young and aged wounds. We found 26% of F4/80^+^ macrophages present in the young Day 3 wounds to be Ki67^+^, while less than 8% of macrophages in the aged Day 3 wounds were Ki67^+^ (**Figures 5G-I**, **Supplementary Figure 5**). We also stained for 53BP1, which is involved in DNA repair and genome protection. The average number of 53BP1^+^ cells was 80% lower in aged wounds compared to young, and these cells constituted a significantly lower proportion of total macrophages present in the tissues (**Figures 5J–L**). These data suggest that ageing-related differences in the macrophage response to wounding result, in part, from alterations to the proliferative potential and genomic stability of these myeloid cells.

### Heightened Inflammation in Aged Macrophages Is Associated With Increased Tissue Damage

**Figure 6:**
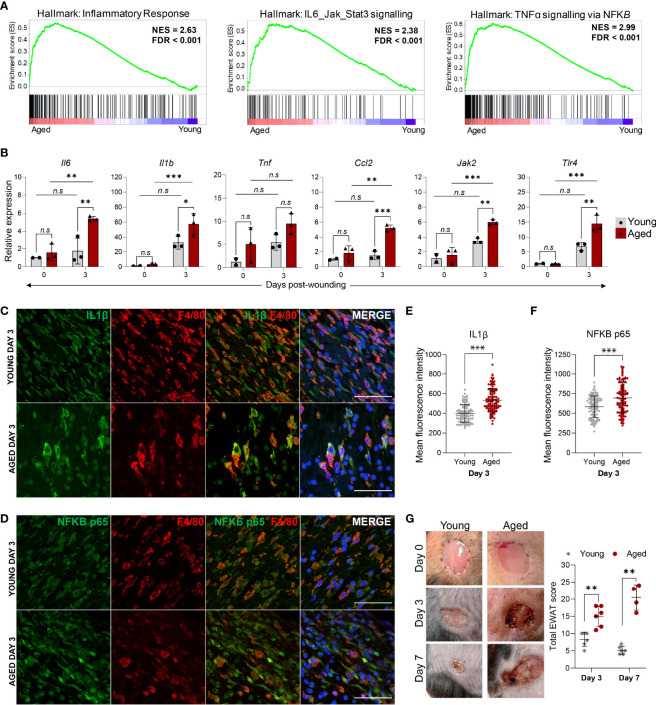
The hyper-inflammatory response in macrophages from aged wounds is correlated with surrounding tissue damage and poor wound healing. **(A)** GSEA plots illustrate the top enriched pathways in aged wound macrophages based on the hallmark gene set. The normalised enrichment score (NES) and the false discovery rate q-value (FDR) are shown. **(B)** Relative expression of proinflammatory genes *Il6, Il1β, Tnf, Ccl2, Jak2* and *Tlr4* in macrophages sorted from young and aged unwounded skin (Day 0) and Day 3 wounds. Expression levels were normalised to Actb and all groups were normalised to young Day 0. n=2-3 mice per group. Bars show mean ± SD. *p<0.05, **p<0.01, ***p<0.001, *n.s*, *not significant*, two-way ANOVA with a Bonferroni multiple comparison test. **(C–D)** Representative images of F4/80 + IL1B or NFκB p65 staining abundance in young and aged Day 3 wounds. 100 cells were analysed from a total of 5-6 FOVS of the wound centre from 3 mice. Scale bar= 50µm. **(E, F)** Mean fluorescence intensity of IL1B or NFκB p65 in F4/80^+^ cells from young and aged Day 3 wounds. Data are shown as mean ± SD. ***p<0.001, unpaired t-test. **(G)** (left) Photographic images of young and aged wounds 0, 3 and 7 days post showing damage of surrounding tissue in aged but not young wounds. (right) Total experimental wound assessment tool score (EWAT score) per mouse in young vs aged mice 3 and 7 days post wounding. **p<0.01, Multiple Mann Whitney U Tests.

In addition to the down-regulation of proliferation processes, macrophages isolated from Day 3 wounds of aged animals were characterised by a prominent elevation of inflammation-related genes. Consistent with the KEGG pathway analysis, GSEA underscored the enrichment of genes involved in the inflammatory response, IL6-Jak-Stat3 signalling and TNF signalling *via* NF-κB pathways in the aged macrophages (**Figure 6A**). We performed qRT-PCR validation of upregulated inflammatory response genes in the pathways that were most significantly altered in the aged using FACS-sorted macrophages from the unwounded skin (Day 0) and Day 3 wounds of young and aged animals. In unwounded skin, no significant differences in these pro-inflammatory factors were detected between the young and aged macrophages, though the average expression of *Il1b*, *Il6*, *Tnf* and *Ccl2* were discernibly higher in the aged cells (**Figure 6B**). In contrast, a distinct ageing-associated increase in the expression of *Il1b*, *Il6*, *Tnf*, *Ccl2, Jak2*, and *Tlr4* in the aged Day3 wound macrophages was observed. Furthermore, we detected significantly higher increases for these genes between Day 0 and Day 3 macrophages in the aged mice compared to the young mice, indicating that the degree of pro-inflammatory gene activation in macrophages in response to wounding was much greater in the aged animals.

We further corroborated these findings at the single-cell level by immunostaining for and quantifying the levels of IL-1β and NF-κB (p65) in F4/80^+^ macrophages present in the Day 3 wounds (**Figures 6C, D** **)**. The mean staining intensities of IL-1β and NF-κB p65 were significantly higher in aged wound macrophages compared to young (**Figures 6E, F** **)**. Hence, while the number of macrophages is substantially lower in aged wounds, these cells produce an aberrantly activated pro-inflammatory transcriptomic and cytokine profile that could contribute to unresolved inflammation in the wounds. Indeed, we observed that aged wounds were characterised by redness, exudate, poor scab formation, and aggravated surrounding tissue damage at Day 3 post-wounding that did not improve or became exacerbated by Day7 (**Figure 6G** **, Supplementary Figure 6**),. We assessed the degree of tissue damage using an experimental wound assessment tool (EWAT) developed by Lima et al. (**Supplementary Table 1**) (16). The total EWAT score for each mouse was calculated to assess the degree of tissue damage (lowest score of 0 indicates no damage; highest score of 36 indicates maximum possible tissue damage). We found that aged wounds had a higher total tissue damage EWAT score on both Days 3 and 7 post-wounding in comparison to young wounds (**Figure 6G**). Aged wounds had an average damage score of 17 at Day 3 which increased to 20 as wound conditions worsened at Day 7 while the young wound damage scores averaged at 8 and improved to 5 by Day 7 (**Figure 6G**). These analyses underscore the poor healing progression of aged wounds and increased inflammation-induced tissue damage that may be related to a heightened inflammatory signature in the macrophages.

## Discussion

In this work, we have analysed ageing-related perturbations to the cellular constitution of cutaneous wounds three and seven days post-injury and investigated the molecular changes underlying the slow healing progression in aged animal models. Our study shows that low numbers of macrophages with reduced proliferative capacity, but a hyper-inflammatory transcriptomic signature is a major feature in poorly healing aged wounds.

Ageing-associated changes in the distribution of macrophages in the skin and wounds have always been a matter of debate due to differences in quantification methods, timeframes of assessments and the challenges of isolating macrophages from these tissues. Past studies have reported contradictory results on how ageing affects the number of macrophages present in cutaneous wounds as healing progresses (14, 15, 37). We employed both flow cytometry and immunohistochemistry analyses to demonstrate that the proportion of macrophages is diminished in Day 3 and Day 7 aged wounds compared to young wounds. We propose that the lower macrophage count in aged animals may result from a cell-autonomous loss of proliferation capacity of these cells. Our *in-vitro* analyses of BMDMs showed that cell cycle progression and checkpoint genes in the aged cells were altered in the same way as macrophages in the wounds of aged mice, despite being removed from the ageing microenvironment. Of note, non-activated BMDMs from unwounded aged mice had also been shown to exhibit cell-intrinsic impaired proliferation, associated with significantly shortened telomeres due and decreased STAT5a phosphorylation (38). Aged human bone marrow cells were also reported to exhibit decreased proliferative capacity, suggesting that cell-intrinsic changes in bone marrow cell proliferation upon ageing may be conserved between humans and mice (39). Reduced numbers of macrophages have also been reported in other tissues such as the lung. A recent study found fewer macrophages in the ageing lung during viral infection in mice and this reduction was ascribed to impaired proliferation of the aged alveolar macrophages (40). It was demonstrated through the adoptive transfer of aged alveolar macrophages into young mice that the tissue microenvironment influenced the proliferative capacity of the cells (40). It remains to be determined if a similar cell-extrinsic effect would be achieved on macrophages in the aged wounds using chimeric mouse models.

We observed a chronic wound-like accumulation of exudates and severe prolonged tissue damage in delayed healing aged wounds, suggestive of a poorly controlled inflammatory response. Macrophages have also been proposed to play a causative role in the delayed healing of chronic wounds (23, 30). Our findings suggest that in the aged mice, inflammation resolution and wound closure is impeded by excessive and sustained activation of inflammatory pathways in the aged wound macrophages, despite a reduced number of these cells in the wounds. This may be related to senescence-related mechanisms. Cellular senescence is associated with the induction of a senescent associated secretory phenotype (SASP) in which elevated production of inflammatory cytokines and chemokines, proteases, and reactive oxygen species results in the death of surrounding cells and damage to the tissue (41–43). Interestingly, non-healing chronic wounds in diabetic mice had been linked to wound macrophages that exhibited a CXCR2 dependent SASP (43).

Our study also highlighted a contribution of F4/80^+^MHCII^lo^ macrophages to the normal inflammatory cell response that is diminished in aged animals. MHCII^lo^ and MHCII^hi^ macrophage subsets are phenotypically and functionally distinct (8, 44–46). MHCII^lo^ macrophages are increased early during an inflammatory response and the reduction of these cells was correlated to increased chemokine secretion and impaired healing upon injury in the lung and heart (44, 47, 48). Beyond the scope of this study, it will be useful, in future experiments, to characterise differential contributions of MHCII^lo^ and MHCII^hi^ macrophage subtypes, through progressive stages of the inflammatory response, to cutaneous wounding healing in young and aged mice with the use of MS4A3 monocyte-lineage tracing mouse models (49).

Though it remains to be investigated if the aberrantly heightened inflammatory responses in aged wound macrophages can be specifically addressed to improve wound recovery, studies in chronic wounds have shown some promising results. Prior work has shown that the rate of healing in chronic wounds can be improved by reducing inflammation through sustained expression and treatment with transcription factor HOXA3 or blockade of regulatory receptors such as the interleukin 1 receptor (50–52). Altered macrophage activities have been previously linked to an ageing-associated elevation of pro-inflammatory factors leading to disease formation in response to injury in organs such as the brain, lung, liver and bone (53–56). Notably, healing outcomes and disease resolution in the old mice could be improved by small-molecule inhibition of macrophage infiltration and inflammatory activities (54, 55). In addition, studies have shown that small-molecule inhibition of the NLRP3 inflammasome in mice resulted in decreased IL1B expression and conferred protection from ageing-related neurodegeneration (57, 58). These data suggest that poor wound repair outcomes associated with both extrinsic and intrinsic ageing-related changes to macrophage behaviour may be regulated by targeting altered inflammatory pathways.

In conclusion, our study has demonstrated that changes in macrophage distribution and function affect the progression of wound healing in female aged mice and provided new insight into functional pathways that can be targeted to aid wound recovery in the elderly. In the future, it will be useful to explore gender-related differences in wound healing responses upon ageing. As macrophages are known to be a heterogeneous population, future single-cell studies of young and aged wound macrophages would reveal the impact of ageing on different functional macrophage subtypes and facilitate the development of more targeted therapeutic approaches.

## Data Availability Statement

The datasets presented in this study can be found in online repositories. The name of the repository and accession number can be found below: NCBI Gene Expression Omnibus; GSE199763.

## Ethics Statement

The animal study was reviewed and approved by A*STAR IACUC, Singapore and the University of Manchester Ethical Review Committee.

## Author Contributions

Conceptualization: KM, CL, and MR. Formal analysis: CD, CL, and KM. Funding acquisition: CL, KM, and MR. Investigation: CD, CL, KM, YO, KW, and SK. Methodology: KM, CL, and CD. Resources: CL, KM, MR, BJ, and JG. Supervision: CL, KM, and MR; Visualization: CD, CL, and TT. Writing – original draft: CD and CL. Writing – review & editing: CD, CL, KM, and MR.

## Funding

The work was supported by the A*STAR IAF-PP Program H17/01/a0/004 to CL and MICRA Seedcorn Award AA17533 to KM and MR. KM is also supported by the British Heart Foundation RM/17/2/33380. CD is supported by the A*STAR Research Attachment Programme and the Faculty of Biology, Medicine and Health at the University of Manchester. KW was supported by EPSRC and MRC CDT in Regenerative Medicine studentship EP/L014904/1. JG is supported by a Kennedy Trust for Rheumatology Research Senior Fellowship.

## Conflict of Interest

The authors declare that the research was conducted in the absence of any commercial or financial relationships that could be construed as a potential conflict of interest.

## Publisher’s Note

All claims expressed in this article are solely those of the authors and do not necessarily represent those of their affiliated organizations, or those of the publisher, the editors and the reviewers. Any product that may be evaluated in this article, or claim that may be made by its manufacturer, is not guaranteed or endorsed by the publisher.

## Abbreviations

BMDMS, Bone marrow-derived macrophages; GSEA, Gene set enrichment analysis; IFNγ, Interferon-gamma; LPS, Lipopolysaccharide; MHCII, Major histocompatibility complex II; TNF, Tumour necrosis factor.
