# Supplementary material for: Age-Related Alterations in Macrophage Distribution and Function Are Associated With Delayed Cutaneous Wound Healing
Source: Front Immunol. 2022 Jul 8;13:943159. doi: 10.3389/fimmu.2022.943159 (PMC9304927; doi:10.3389/fimmu.2022.943159)
Supplement: Supplementary file 1 [file DataSheet_1.docx]

Supplementary Material

#
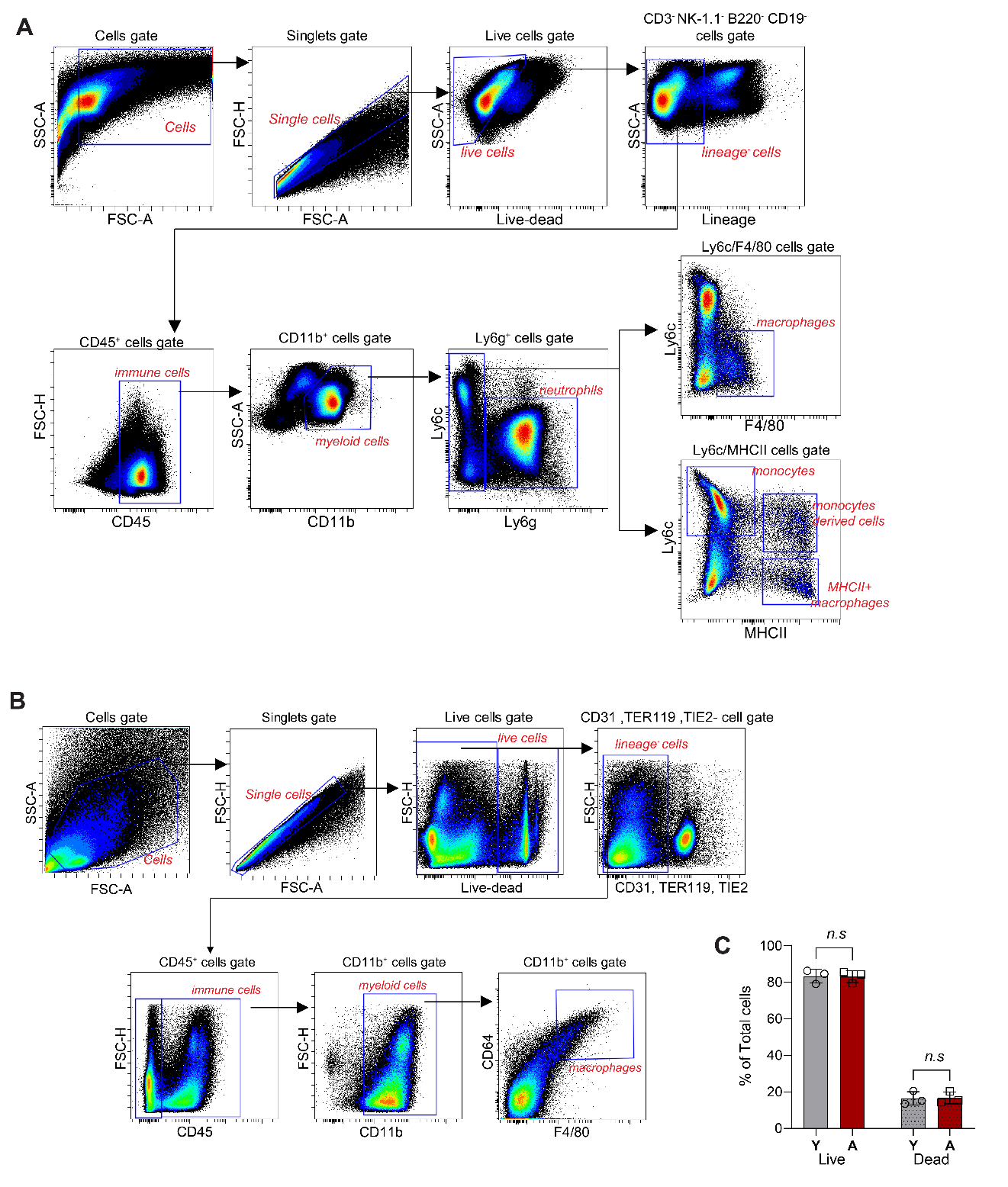


**Supplementary Figure S1. Manual gating strategies used to validate populations identified by viSNE in young and aged mice. (A)** Manual gating strategy showing myeloid subset populations validating viSNE clusters**. (B)** Manual gating strategy used for FACS sorting of macrophages isolated from wounds for RNA sequencing. (C) Percentage live and dead populations are shown. n=3 per group, two-way ANOVA with a Bonferroni multiple comparisons test.

**
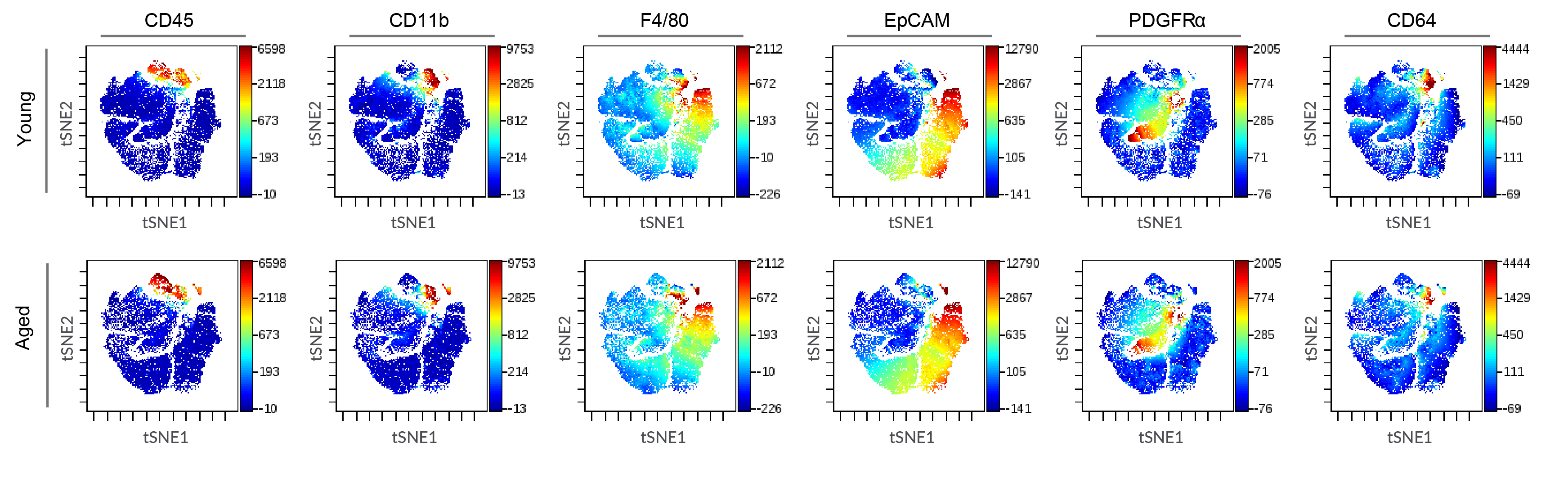
**

**Supplementary Figure S2. Expression pattern of cell markers defining distinct populations in young and aged skin.** Individual viSNE maps were generated using 6 markers. viSNE plots are coloured by expression levels of CD45, CD11b, F4/80, EpCAM, PDGFRα and CD64 in young and aged skin cells. 15000 cells were analysed per group.


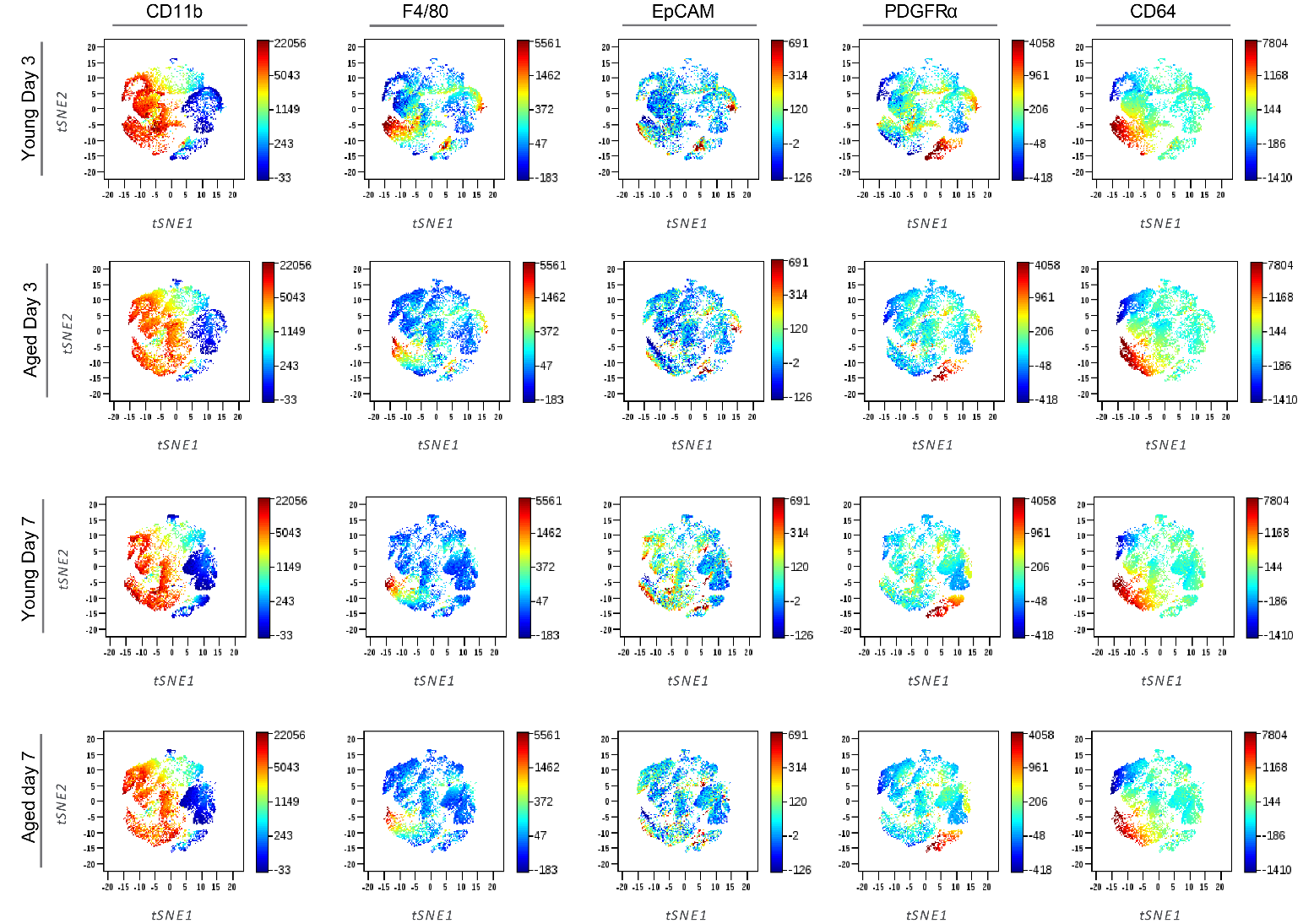
**Supplementary Figure S3. Individual distribution of cell type-specific markers in young and aged wounded mice.** Individual viSNE maps generated using 5 markers used to define skin cell population in young and aged mice 3 and 7 days post-wounding. viSNE plots are coloured by expression levels of CD11b, F4/80, EpCAM, PDGFRα and CD64. 10000 cells were assessed per group.


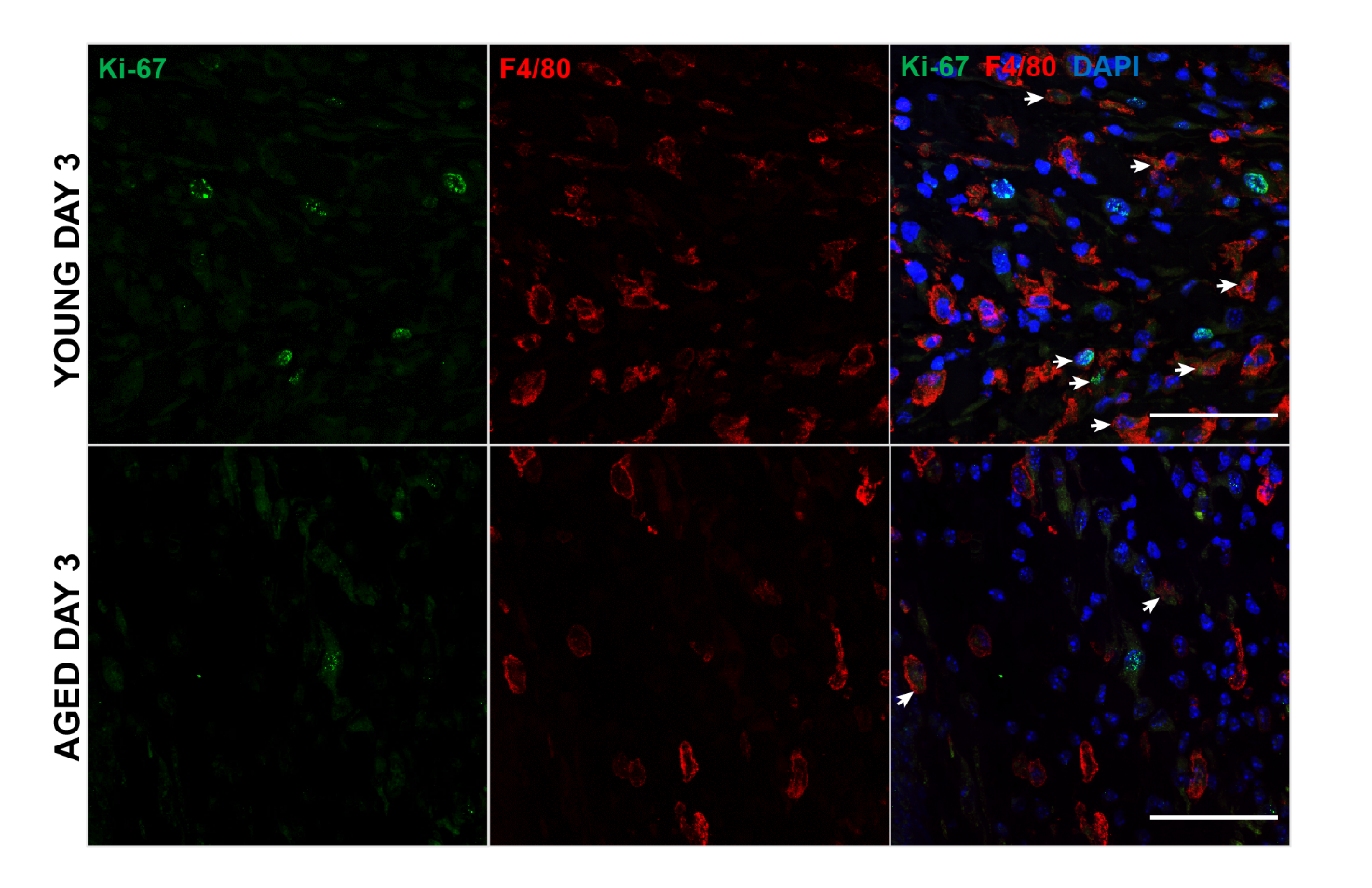
**
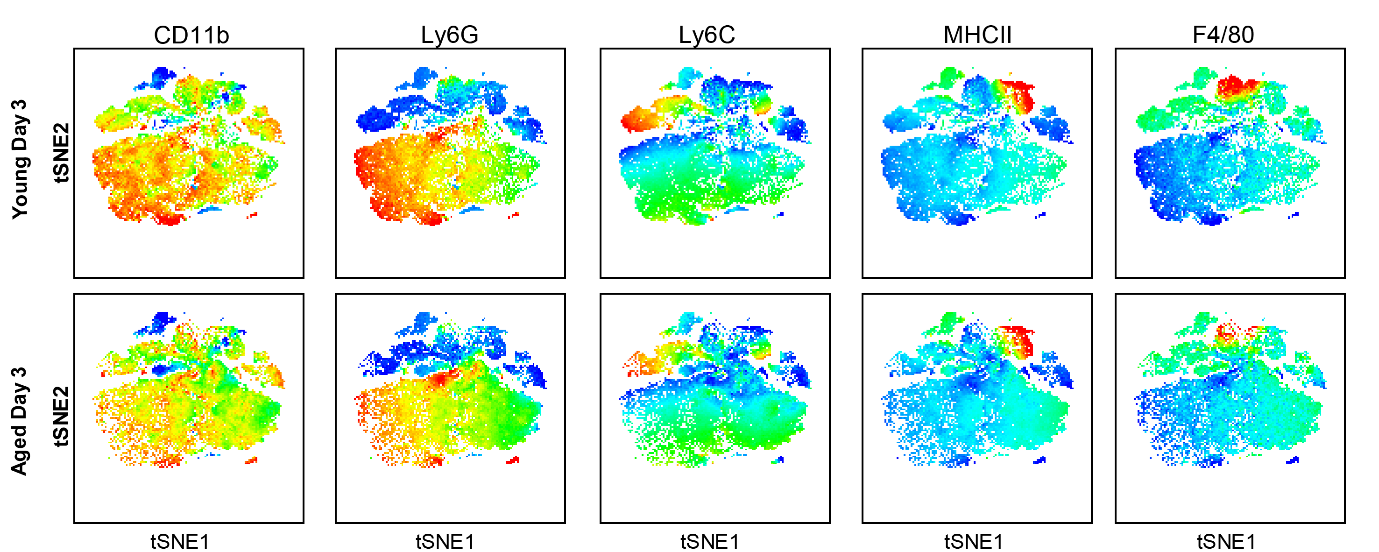
Supplementary Figure S4. Expression pattern of cell markers defining distinct populations in young and aged Day 3 wounds.** Individual viSNE maps were generated using 5 distinct markers. viSNE plots are coloured by expression levels of, CD11b, Ly6G, Ly6C, MHCII, and F4/80 in young and aged skin cells. 25000 cells were analysed per group.

**Supplementary Figure S5. Representative images of F4/80 and Ki-67 staining in young and aged Day 3 wounds.** Arrowheads indicate F4/80^+^ Ki-67^+^ double-positive cells. Scale bar= 50µm.


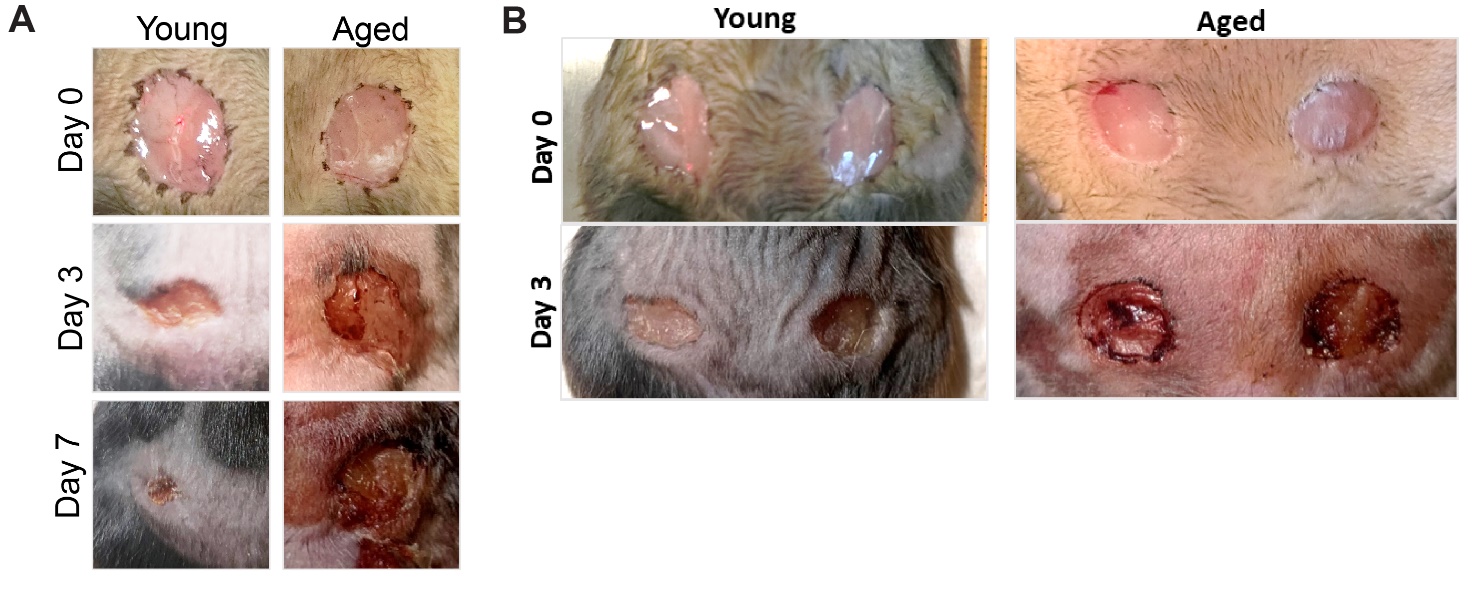


**Supplementary Figure S6. Comparison of wound phenotype between young and aged mice. (A-B)** Representative photographic images of young and aged wounds 0, 3 and 7 days post showing inflammation associated damage of surrounding tissue in aged but not young wounds**.**

| **Wound Parameter** | **Score** |
| --- | --- |
| 1. Area (cm^2^) | 0 – no ulcer  1 – < 0.01 cm^2^  2 – 0.01 to 0.09 cm^2^  3 – 0.10 to 0.29 cm^2^  4 – 0.30 to 0.69 cm^2^  5 – 0.70 to 1.09 cm^2^  6 – 1.10 to 2.09 cm^2^  7 – 2.10 to 3.09 cm^2^  8 – 3.10 to 4,09 cm^2^  9 – 4.10 to 8.09 cm^2^  10 – 8.10 to 11.99 cm^2^  11 – > 12 cm^2^ |
| 1. Exudate Type | 0 – none  1 – bloody  2 – serosanguineous  3 – serous  4 - purulent |
| 1. Exudate amount | 0 – none  1 – light  2 – moderate  3 – heavy |
| 1. Tissue type | 0 – Epithelial tissue  1 – granulation tissue or ischemic skin  2 – slough  3 – eschar |
| 1. Necrotic Tissue type | 0 – none visible  1 – white/gray/yellow non adherent slough  2 – white/gray/yellow loosely adherent slough  3 – adherent, soft, black or brown eschar  4 – firmly adherent, hard, black or brown eschar |
| 1. Necrotic tissue amount | 0 – None visible  1 – < 25% of wound bed covered  2–25% to 50% of wound bed covered  3 – > 50% and < 75% of wound bed covered  4 – 75% to 100% of wound bed covered |
| 1. Granulation tissue | 0 – epithelial tissue  1 – 50% to 100%  2 – < 50% of wound  3 – obscured by necrosis |
| 1. Skin colour surrounding the wound | 0 – Normal for animal's species  1 – Red or erythema  2 – White or pale or gray pallor  3 – Dark red or purple  4 – Black |

**Supplementary Table 1: Experimental wound assessment tool by Lima et al. 2018 (EWAT)**

**Supplementary Table 2:** List of antibodies used for low-parameter flow analyses and FACS sorting

| **Antibody** | **Fluorophore** | **Clone** | **Catalogue number** | **Supplier** |
| --- | --- | --- | --- | --- |
| antimouse-CD326 (EpCAM) | APC Efluor 780 | G8.8 | 47−5791−80 | EBiosciences |
| antimouse-CD11b | BV650 | M1/70 | 101249 | Biolegend |
| antimouse-CD45 | BV510 | 30-F11 | 103138 | Biolegend |
| DAPI | DAPI |  | D1306 | Life Technologies |
| antimouse-CD140a (PDGFRα) | PE/Dazzle™ 594 | APA5 | 135922 | Biolegend |
| anti-mouse- CD31 | PE | 390 | 102408 | Biolegend |
| antimouse- TER119 | PE | Ter-119 | 116223 | Biolegend |
| antimouse- CD202b (TIE2) | PE | TEK4 | 12-5987-82 | eBiosciences |
| anti-mouse F4/80 | PE/Cy7 | BM8 | 123114 | Biolegend |
| anti-mouse CD64 | APC | X54-5/7. 1 | 139306 | Biolegend |

**Supplementary Table 3:** List of antibodies used for high dimensional analyses

| **Antibody** | **Fluorophore** | **Clone** | **Catalogue number** | **Supplier** |
| --- | --- | --- | --- | --- |
| anti-mouse MHC II (IA, IE) | APC | M5/114.15.2 | 107622 | Biolegend |
| anti-mouse CD45 | Brilliant Violet 510 | 30-F11 | 48-0451-82 | E-bioscience |
| anti-mouse CD11b | Brilliant Violet 605 | M1/70 | 101237 | Biolegend |
| anti-mouse Ly6C | Brilliant Violet 711 | HK1.4 | 128037 | Biolegend |
| anti-mouse ZOMBIE Live/Dead | Ultra-Violet | n/a | 423107 | Biolegend |
| anti-mouse Ly6G | PE-CF594 | 1A8 | 562700 | BD Biosciences |
| anti-mouse F4/80 | PE/Cy7 | BM8 | 123114 | Biolegend |
| anti-mouse CD16/CD32 | FC BLOCK | 2.4G2 | 553142 | BD Biosciences |
| anti-mouse CD3 | APC-Cy7 | 17A2 | 47-0032 | E-bioscience |
| anti-mouse NK-1.1 | APC-Cy7 | PK136 | 47-5941 | E-bioscience |
| anti-mouse CD45R/B220 | APC-Cy7 | RA3-6B2 | 47-0452-82 | E-bioscience |
| anti-mouse CD19 | APC-Cy7 | 1D3 | 47-0193 | E-bioscience |

**Supplementary Table 4.** List of primers used for qRT-PCR

| **Primer** | **Forward** | **Reverse** |
| --- | --- | --- |
| *Mcm6* | CCTGAGAGAAACACGCTGGT | GGTCTTCAAGGCTCGACACA |
| *Gins2* | ACAGAAGTGCCGCCTGTTAC | TGATCTCCATGTAGTGCGGG |
| *Rpa1* | TGGGACACAGTCCAAAGTGG | CCAGGTGCGGATCTGACTTT |
| *Xrcc6* | AACACCGGCAGTCTACTCCT | TGGCTCATCAAACCGCTTCA |
| *Parp1* | GCGGAGAAGACATTGGGTGA | ACCATCTTCTTGGACAGGCG |
| *Cdk1* | CTTCAGGATCTTCAGAGCTCTGG | AAAGTACGGGTGCTTCAGGG |
| *Ccnb1* | AGAGGTGGAACTTGCTGAGCCT | GCACATCCAGATGTTTCCATCGG |
| *Il6* | GACAAAGCCAGAGTCCTTCAGA | AGGAGAGCATTGGAAATTGGGG |
| *Il1β* | AAGTTGACGGACCCCAAAAGA | AAGCTGGATGCTCTCATCAGG |
| *Tnf* | CTGTAGCCCACGTCGTAGC | TTTGCTACGACGTGGGCTAC |
| *Ccl2* | TGGAGCATCCACGTGTTGG | TGTAGCTCTCCAGCCTACTCA |
| *Jak2* | GGCGACGGGAACAAGATGT | CAGGCCATTCCCATCTAGAGC |
| *Tlr4* | TCAGAACTTCAGTGGCTGGA | AGAGGTGGTGTAAGCCATGC |
| *Tbp* | TTTGGCTAGGTTTCTGCGGT | ACGTCTTCAATGTTCTGGGT |
| *Actb* | AAGGCCAACCGTGAAAAGAT | GTGGTACGACCAGAGGCATAC |
| *Hist2aa2* | GCCCGCGTCTCTGTGATA | CCGTGACACAACTCTTTATCTGA |

**Suppl. Table 5.** Primer pool for amplification with Prelude PreAmp master mix

| Primer ID | *Gene* | Forward | Reverse |
| --- | --- | --- | --- |
| 1 | *Mcm2* | ACTCATTGGTGATGGCATGGA | CAATCCCTCGGCCTCGTAGA |
| 2 | *Rad51* | TACATTGACACCGAGGGCAC | GTGTTGAACCCTCGCGCATA |
| 3 | *Msh2* | TATTAACCAGCTCCCCAGCG | AAACTGCCAACAACAGTGCC |
| 4 | *Xrcc6* | AACACCGGCAGTCTACTCCT | TGGCTCATCAAACCGCTTCA |
| 5 | *Rpa1* | TGGGACACAGTCCAAAGTGG | CCAGGTGCGGATCTGACTTT |
| 6 | *Dpy30* | AAGGTGGATCTACAGTCCTTGC | CGATGGGATTTGGTGGTC |
| 7 | *Cdk1* | CTTCAGGATCTTCAGAGCTCTGG | AAAGTACGGGTGCTTCAGGG |
| 8 | *Ccnb1* | AGAGGTGGAACTTGCTGAGCCT | GCACATCCAGATGTTTCCATCGG |
| 9 | *Cdc20* | ATTTGGAACGTCTGCTCAGGG | GTGGGGAGACCAGAGGATGG |
| 10 | *Ccna2* | CCCGGAGCAAGAAAACCACT | TCATTAACGTTCACTGGCTTGT |
| 11 | *Aurkb* | AGATTGCAGACTTTGGCTGGT | ATTTCATTATGCATGCGCCCC |
| 12 | *Wwtr1* | TAGGATGCGTCAAGAGGAGC | GCCATGGTCTCGGTTTCCAT |
| 13 | *Il6* | GACAAAGCCAGAGTCCTTCAGA | AGGAGAGCATTGGAAATTGGGG |
| 14 | *Nlrp3* | TTCCCCTTTATTTGTACCCAAGG | CAGACGTATGTCCTGAGCCA |
| 15 | *Tnf* | CTGTAGCCCACGTCGTAGC | TTTGCTACGACGTGGGCTAC |
| 16 | *Il1b* | AAGTTGACGGACCCCAAAAGA | AAGCTGGATGCTCTCATCAGG |
| 17 | *Stat1* | GTGGGAACGGAAGCATTTGG | ATCGTACAGCTGGTGGACCT |
| 18 | *Irf7* | TGTACGAACTTAGCCGGGAG | GTACTGCAGAACCTGTGTGGG |
| 19 | *Ccl2* | TGGAGCATCCACGTGTTGG | TGTAGCTCTCCAGCCTACTCA |
| 20 | *Nfkb1* | CTGCTCAGGTCCACTGTCTG | TGTCACTATCCCGGAGTTCA |
| 21 | *Nfkb2* | CGGCAGTCTCCTTCGTAGTT | ACCTTTCAGCAGCCTGACTC |
| 22 | *Tlr4* | TCAGAACTTCAGTGGCTGGA | AGAGGTGGTGTAAGCCATGC |
| 23 | *Cx3cr1* | CTGCAGAAGTTCCCTTCCCA | TAACAGGCCTCAGCAGAATCG |
| 24 | *Nfkbia* | GGCCAGTGTAGCAGTCTTGA | CGTGGATAGAGGCTAGGTGC |
| 25 | *Jak2* | GGCGACGGGAACAAGATGT | CAGGCCATTCCCATCTAGAGC |
| 26 | *Hdac10* | TTCCAGGATGAGGATCTTGC | ACATCCAATGTTGCTGCTGT |
| 27 | *Kdm5c* | ACAGTAAGCGGCACCTAACC | GTGGCACAGTACAGACTGCT |
| 28 | *Jarid2* | GCAGTGGCGAAGGTTTGCAT | TCCGACTTTTCTTCCTCTTCCC |
| 29 | *L3mbtl3* | AAGACGCCCCGACTTCTC | GGGTACCCCAGTCTTCTCTGA |
| 30 | *Gadd45a* | AGAGCAGAAGACCGAAAGGA | CGTAATGGTGCGCTGACTC |
| 31 | *Jade3* | GCAAGTTGAAGACAGGGGCT | GCACAGGTGACATGAAAGGC |
| 32 | *Xbp1* | AACACGCTTGGGAATGGACA | ACATAGTCTGAGTGCTGCGG |
| 33 | *Chaf1* | TGGATTGCAAAGACAGACCCG | TTCAGGCGTTTGAAGGGCAA |
| 34 | *Asf1b* | TTGATCAGATCCTAGATTCAGTGC | GAATGAGGGATGGGTTTGG |
| 35 | *Setdb2* | GCCAAGTGAGAGGACAAAGATT | CAGAGATTTGGACAGCAACTATGA |
| 36 | *Smyd2* | CGTGTTTGAGGACAGCAATG | GGAGTACACGGGGTAGTGCT |
| 37 | *Elp3* | CGCAAGGGAGGAAGTGGATT | ACCAGCTGCTTGATAACGTCT |
| 38 | *Bh1he41* | CTTGAAAGCGCTAACAGCCT | CGAGTGGAACGCATCCAAGT |
| 39 | *Tbp* | TTTGGCTAGGTTTCTGCGGT | ACGTCTTCAATGTTCTGGGT |
| 40 | *Gapdh* | AGCTTGTCATCAACGGGAAG | TTTGATGTTAGTGGGGTCTCG |
| 41 | *Actb* | AAGGCCAACCGTGAAAAGAT | GTGGTACGACCAGAGGCATAC |
| 42 | *kdm6b* | CTGTACAGACCCCCGGAAC | TGGTGGAGAAAAGGCCTAAGT |
| 43 | *Ccnd1* | TCTTTCCAGAGTCATCAAGTGTG | CTTGGGGTCGACGTTCTG |
| 44 | *Cdk4* | CATACCTGGACAAAGCACCTCC | GAATGTTCTCTGGCTTCAGGTCC |
| 45 | *Cdk2* | CACAGCCGTGGATATCTGG | CATGGTGCTGGGTACACACT |
| 46 | *Ccne1* | CTGAGAGATGAGCACTTTCTGC | TGGAGCTTATAGACTTCGCACA |
